# Meiotic MCM Proteins Promote and Inhibit Crossovers During Meiotic Recombination

**DOI:** 10.1534/genetics.119.302221

**Published:** 2019-04-26

**Authors:** Michaelyn Hartmann, Kathryn P. Kohl, Jeff Sekelsky, Talia Hatkevich

**Affiliations:** * Curriculum in Genetics and Molecular Biology, University of North Carolina, Chapel Hill, North Carolina 27599; † Department of Biology, Winthrop University, Rock Hill, South Carolina 29733; ‡ Department of Biology, University of North Carolina, Chapel Hill, North Carolina 27599; § Integrative Program in Biological and Genome Sciences, University of North Carolina, Chapel Hill, North Carolina 27599

**Keywords:** *Drosophila*, meiotic recombination, meiosis, crossover, mei-MCM

## Abstract

Crossover formation as a result of meiotic recombination is vital for the proper segregation of homologous chromosomes at the end of meiosis I. In many organisms, crossovers are generated through two crossover pathways: Class I and Class II. To ensure accurate crossover formation, meiosis-specific protein complexes regulate the degree to which each pathway is used. One such complex is the mei-mini-chromosome maintenance (MCM) complex, which contains MCM and MCM-like proteins REC (ortholog of Mcm8), MEI-217, and MEI-218. The mei-MCM complex genetically promotes Class I crossovers and inhibits Class II crossovers in *Drosophila*, but it is unclear how individual mei-MCM proteins contribute to crossover regulation. In this study, we perform genetic analyses to understand how specific regions and motifs of mei-MCM proteins contribute to Class I and II crossover formation, and distribution. Our analyses show that the long, disordered N-terminus of MEI-218 is dispensable for crossover formation, and that mutations that disrupt REC’s Walker A and B motifs differentially affect Class I and Class II crossover formation. In *rec* Walker A mutants, Class I crossovers exhibit no change but Class II crossovers are increased. However, in *rec* Walker B mutants, Class I crossovers are severely impaired and Class II crossovers are increased. These results suggest that REC may form multiple complexes that exhibit differential REC-dependent ATP-binding and -hydrolyzing requirements. These results provide genetic insight into the mechanisms through which mei-MCM proteins promote Class I crossovers and inhibit Class II crossovers.

TO reestablish the diploid genome upon sexual fertilization, the genome of progenitor germ cells must be successfully reduced by one-half through meiosis. Accurate reduction of the genome at the end of meiosis I requires crossover formation between homologous chromosomes during meiotic recombination. Meiotic recombination is initiated by the formation of multiple double-strand breaks (DSBs); the majority of meiotic DSBs are repaired as noncrossovers, while a selected subset is repaired as crossovers between homologs [reviewed in Lake and Hawley (2012)].

Two distinct types of meiotic crossovers have been described: Class I and Class II. First defined in budding yeast (de los Santos *et al.* 2003), Class I and Class II crossovers exist in most sexually reproducing organisms, but the relative proportions of each crossover type vary among organisms (Hollingsworth and Brill 2004). In *Drosophila*, most—if not all—crossovers are generated through the Class I pathway (Hatkevich *et al.* 2017), as shown through their dependence on the putative catalytic unit of the Class I meiotic resolvase MEI-9 (Sekelsky *et al.* 1995; Yildiz *et al.* 2002) and their display of crossover interference (Hatkevich *et al.* 2017). Most crossovers in *Drosophila* are also dependent upon a group of (mini-chromosome maintenance) MCM or MCM-like proteins, called the mei-MCM complex (Baker and Carpenter 1972; Grell 1978; Liu *et al.* 2000; Kohl *et al.* 2012).

The mei-MCM complex consists of REC (the *Drosophila* ortholog of MCM8), MEI-217, and MEI-218. REC appears to be a *bona fide* MCM protein, based on conservation of both the N-terminal MCM domain and the C-terminal AAA+ ATPase domain, which includes Walker A and B boxes that bind and hydrolyze ATP (Figure 1A). In contrast, MEI-217 and MEI-218 are highly divergent MCM-like proteins, and together resemble one full MCM protein. MEI-217 is structurally similar to the MCM N-terminal domain, though this similarity is not detected in basic local alignment search tool or conserved domain searches (Kohl *et al.* 2012). The C-terminus of MEI-218 has a domain related to the AAA+ ATPase domain, but key residues are not conserved, including the Walker A and B motifs, which are critical for binding and hydrolyzing ATP, respectively (Iyer *et al.* 2004) (Figure 1B). Because key residues in the Walker A and B motifs are not conversed, MEI-218 may not exhibit ATPase activity or it may exhibit partial function. In addition, MEI-218 has a long N-terminal extension that is poorly conserved and is predicted to be disordered. The function of this region is unknown, but gene swap studies suggest that it may contribute to differences in the recombination landscape among *Drosophila* species (Brand *et al.* 2018). For further analysis and details regarding the evolution of the mei-MCM complex, see Supplemental Material, Figures S1–S3.

**Figure 1:**
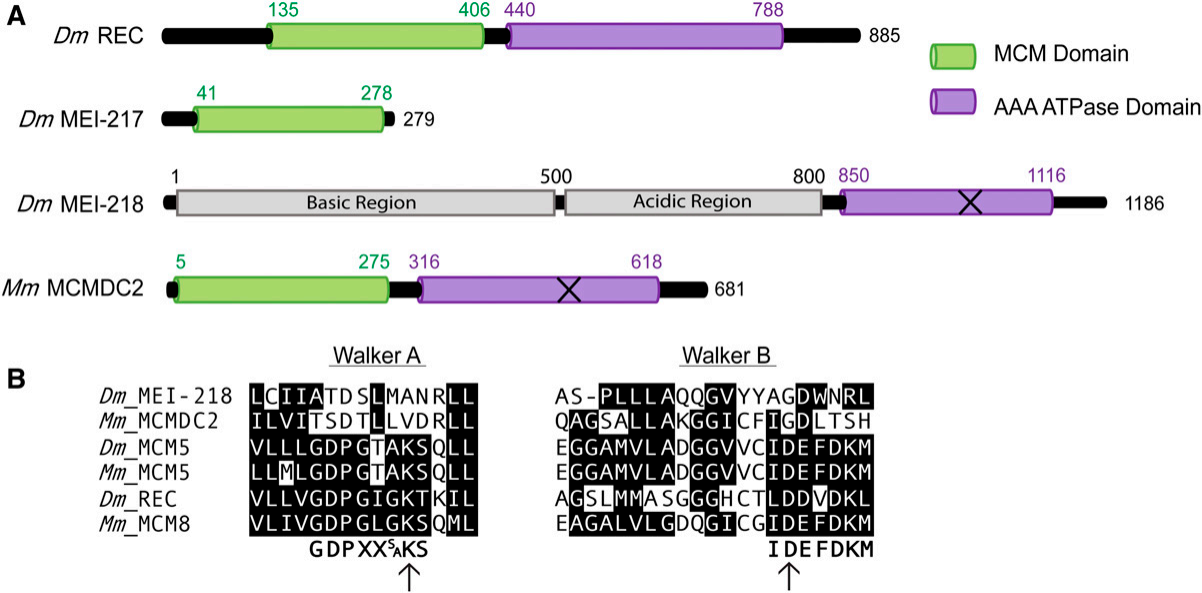
MCM protein structures and alignments. (A) Structural domains of *Dm* REC, MEI-217, and MEI-218 and *Mm* MCMDC2. Structural domains identified using PHYRE 2 (Kohl *et al.* 2012). “MCM domain” corresponds to protein data bank ID #c2vl6C and the AAA ATPase domains identified correspond to protein data bank ID #d1g8pa. The “X” on *Dm* MEI-218 and *Mm* MCMDC2 represent predicted inactive AAA ATPase domains. (B) Consensus sequences for Walker A (Walker *et al.* 1982) and Walker B motifs (Forsburg 2004). Identical or conserved amino acids are denoted with black background. Arrows denote the conserved catalytic residues. *Dm*, *D. melanogaster*; ID, identifier; MCM, mini-chromosome maintenance; *Mm*, *M. musculus*.

While most crossovers are generated through the Class I pathway in wild-type *Drosophila* and are mei-MCM-dependent, mutants that lack the Bloom syndrome helicase (Blm) generate only Class II crossovers, based on their independence of MEI-9 and lack of the patterning (*e.g.*, interference) that is associated with Class I crossovers (Hatkevich *et al.* 2017). Blm is an ATP-dependent 3′–5′ helicase that exhibits vital anticrossover functions in both meiotic and somatic DSB repair [reviewed in Hatkevich and Sekelsky (2017)]. Interestingly, mutations in *mei-MCM* and *Blm* genes genetically interact. In *Blm* mutants, crossovers are reduced by 30% but in a *Blm rec* double mutant, crossovers are significantly increased compared to wild-type (Kohl *et al.* 2012). This suggests that the mei-MCMs may function to inhibit crossovers within the Class II pathway, in addition to their role in promoting crossovers in the Class I pathway.

While the mei-MCMs function as a complex, little is known about how individual mei-MCMs contribute to Class I and II crossover regulation. Here, we investigate specific features of MEI-218 and REC to better understand how these proteins contribute to meiotic recombination. We find that the N-terminus of MEI-218 is dispensable for crossover formation and general crossover distribution. By mutating key residues in REC’s Walker A and B motifs (*rec^KA^* and *rec^DA^*, respectively), we find that *rec^KA^* mutants exhibit no Class I crossover defect, while Class II crossovers are significantly increased. Surprisingly, *rec^DA^* mutants exhibit a severe decrease in Class I crossovers and a significant increase in Class II crossovers. Our results suggest that the mei-MCMs function in multiple roles and may complex in a variety of configurations to properly regulate crossover formation.

## Materials and Methods

### Drosophila stocks

Flies were maintained on standard medium at 25°. Some mutant alleles have been previously described, including *mei-9^a^* (Baker and Carpenter 1972; Yildiz *et al.* 2004), *mei-218^1^* and *mei-218^6^* (Baker and Carpenter 1972; McKim *et al.* 1996), *Blm^N1^* and *Blm^D2^* (McVey *et al.* 2007), and *rec^1^* and *rec^2^* (Grell 1978; Matsubayashi and Yamamoto 2003; Blanton *et al.* 2005). The maternal-effect lethality in *Blm^N1^/Blm^D2^* mutants was overcome by the upstream activation sequence (*UAS)*::*GAL4* rescue system, as previously described (Kohl *et al.* 2012).

### Generating mei-218 transgenic alleles

The transgenes for *mei-218*^△^*^N^* and *mei-218^FL^* were constructed by cloning cDNA for *mei-218* into *P*{*attBUASpW*} (Addgene). Full-length *mei-218* included codons 1–1186; the *mei-218*^△^*^N^* transgene included codons 527–1186. Transgenics were made by integrating into a ϕC31 landing site in 2A on the *X* chromosome.

### Generating rec^KA^ and rec^DA^ mutants

Annealed oligonucleotides were inserted into *Bbs*I-digested pU6-BbsI-chiRNA plasmid (Addgene) (*rec^KA^*: 5′-CTTCGCCGAGAAGGGATAGTAAAC-3′ and *rec^DA^*: 5′-CTTCGTTGCAGTGCCTACAATCAG-3′). The resulting plasmids were co-injected with repair template plasmid, consisting of synthesized gBlocks (Integrated DNA Technologies) cloned into pBlueScript plasmid (sequences available on request). Injected larvae were raised to adulthood and their male progeny were crossed to *TM3*/*TM6B* females (Bloomington *Drosophila* Stock Center) to generate stocks, after which DNA was extracted for screening through PCR and restriction digest.

### Nondisjunction assay

*X*-chromosome nondisjunction (NDJ) was assayed by mating virgin females to *y cv v f*/*T(1:Y)B^S^* males. Each cross was set up as a single experiment with 20–50 separate vials. The progeny of each vial were counted separately. Viable NDJ progeny are *XXY* females with Bar eyes, and *XO* males with Bar^+^ eyes and the phenotypes from *y cv v f* chromosome. Total (adjusted) represents the total with inviable exceptional progeny accounted for (*XXX* and *YO*). NDJ rates and statistical comparisons were done as in Zeng *et al.* (2010).

### Crossover distribution assay

Crossover distribution on chromosome 2L was scored by crossing virgin *net dpp^d-ho^ dp b pr cn/+* female flies with the mutant background of interest to *net dpp^d-ho^ dp b pr cn* homozygous males. Each cross was set up as a single experiment with ≥ 25 separate vials scored. The first set of vials was flipped after 3 days of mating into vials of a new batch, although these were counted as one experiment. Batch effects for recombination assays have not been observed in repeated studies for multiple genotypes used in this study (Figure S4). These include wild-type (unpublished data, M. Hartmann), *Blm* (unpublished data, M. Hartmann), *rec* (Blanton *et al.* 2005; Kohl *et al.* 2012), *mei-9* (Sekelsky *et al.* 1995), and *mei-9*; *rec* (Blanton *et al.* 2005). All progeny were scored for parental and recombinant phenotypes. Crossover numbers in flies are shown as cM where cM = (number of crossovers/total number of flies) * 100. χ^2^ tests with Bonferroni correction were performed for each interval. For total cM, Fisher’s exact test was used to compare total crossovers to the total number of flies. Crossover distribution is represented as cM/Mb where Mb is length of the interval without transposable elements (TEs), because crossovers rarely occur within TEs (Miller *et al.* 2016).

### Protein structure and alignment

Structural domains of proteins were determined by using PHYRE 2. All of the MCM regions identified correspond to the protein data bank identifier (ID) #c2vl6C and the AAA ATPase domains identified correspond to protein data bank ID #d1g8pa. Alignment of the Walker A and Walker B motifs (Kohl *et al.* 2012) was done using MEGA 5 with the ClustalW program. Identical and conserved residues are shaded based on groups of amino acids with similar chemical properties.

### Data availability

All data necessary for confirming the conclusions in this paper are included in this article, and in supplemental figures and tables. *Drosophila* stocks and plasmids described in this study are available upon request. Figure S1 illustrates the distribution of MSH4, MSH5, MCM8, MCM9, MEI-217, and MEI-218 in Diptera. Figure S2 illustrates the structure of MEI-217 and MEI-218 in Diptera. Figure S3 shows sequence alignments of MEI-218. Figure S4 compares crossover frequencies in different batches of the same genotype. Figure S5 details the cross scheme of the *mei-218* transgene experiments. Table S1 includes analysis of genetic interval differences between wild-type and *mei-218^FL^*. Table S1 includes analysis of genetic interval differences between *mei-218^FL^* and *mei-218^∆N^*. Table S2 includes the complete data set for calculating NDJ of wild-type, *rec^−^/rec^+^*, and *rec^DA^/+* flies. Table S3 includes all data sets for meiotic crossovers for all genotypes discussed. Supplemental material available at Figshare: https://doi.org/10.25386/genetics.8009426.

## Results and Discussion

### The N-terminus of MEI-218 is dispensable for crossover formation

MCMDC2 is a distantly related member of the MCM family of proteins in mammals that is unique in that the ATPase domain is predicted to be incapable of binding or hydrolyzing ATP. Orthologs in Dipteran insects are further distinguished by having the N-terminal and ATPase-like domains encoded in separate open reading frames (Figure S2). The two polypeptides MEI-217 and MEI-218 interact physically, at least in *Drosophila melanogaster*, presumably reconstituting a single MCM-like protein. MEI-218 is also distinguished by possessing an N-terminal extension of variable length in different species. *D. melanogaster* MEI-218 can be divided into three distinct regions (Figure 1A): an N-terminal tail (residues 1–500), a central acidic region (residues 500–800), and a C-terminal ATPase-related region (residues 850–1116) (Kohl *et al.* 2012; Brand *et al.* 2018). The N-terminal and middle regions are predicted to be disordered (Kohl *et al.* 2012), and are poorly conserved (Figure S3). Results obtained through gene-swap experiments suggest that the N-terminal tail and central region regulate crossover number and distribution within *Drosophila* species (Brand *et al.* 2018).

**Figure 2:**
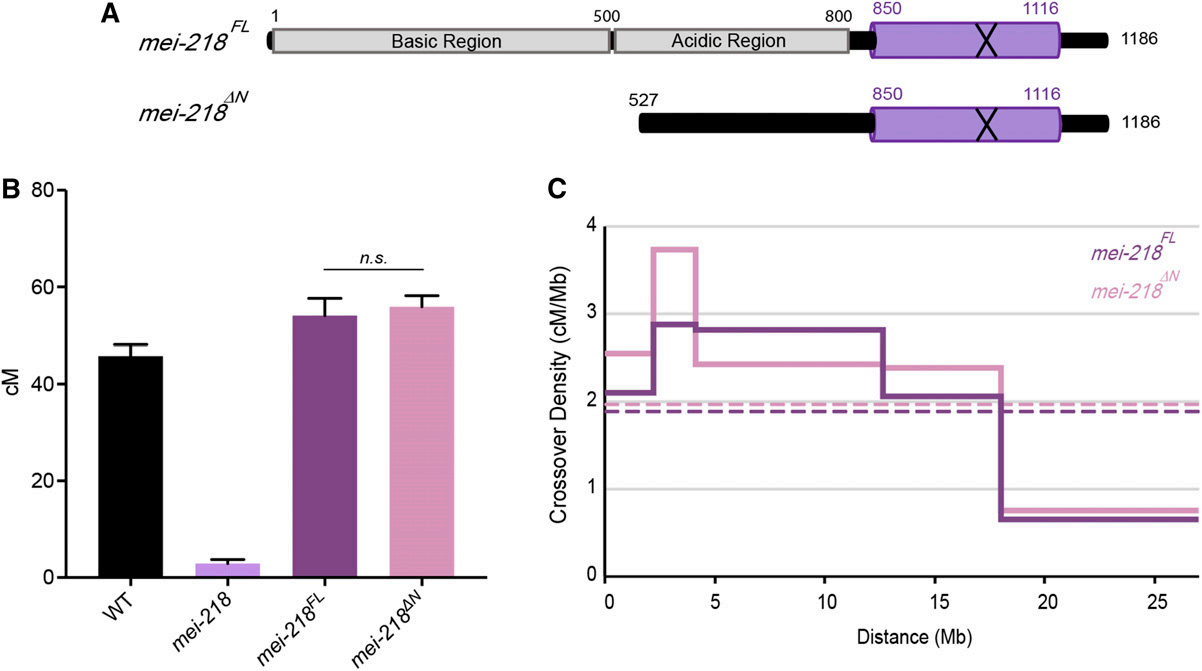
The role of the MEI-218 N-terminus in crossover formation and distribution. (A) Schematic of transgenes for full-length *mei-218* and N-terminal-deleted *mei-218*, in which the first 526 amino acids are absent. (B) Map units of WT (Hatkevich *et al.* 2017), *mei-218* (Kohl *et al.* 2012), *mei-218^FL^*, and *mei-218*^∆N^. Map units represented as centimorgans. Error bars indicate 95% C.I.s. (*P* = 0.61). (C) Crossover distribution (solid lines) of *mei-218^FL^* and *mei-218*^∆N^ represented as cM/Mb. “Megabase” represents the measured distance of the defined interval, excluding the centromere, pericentromeric heterochromatin, and transposable elements. Dotted lines represent mean crossover density across 2L. Figure S5 details the cross scheme of *mei-218* transgene experiments. Refer to Tables S1 and S3 for complete data sets. n.s., not significant; WT, wild-type.

To genetically examine the function of the N-terminus of MEI-218, we compared the functions of a transgene that expresses a truncated form of MEI-218 that lacks the N-terminal 526 amino acids (*mei-218*^△^*^N^*) to a matched full-length transgene (*mei-218^FL^*) (Figure 2A). Due to the relatively high conservation among *Drosophila* species, the middle region of MEI-218 was retained for this experiment (Figure S3). Using the *UAS*/*GAL4* system (Duffy 2002), we expressed both constructs in *mei-218* null mutants using the germline-specific *nanos* promoter and measured crossovers along five adjacent intervals, which span most of 2L and part of 2*R* (Figure S4; for simplicity, we refer to this chromosomal region as 2L.)

In wild-type females, the genetic length of 2L is 45.8 cM (Hatkevich *et al.* 2017) (Figure 2B), whereas *mei-218* mutants exhibit a severe decrease in crossovers, with a genetic length of 2.92 cM (Kohl *et al.* 2012). Expression of *mei-218^FL^* in *mei-218* mutants (*mei-218^FL^*) fully rescues the crossover defect, exhibiting a genetic length of 54.1 cM. Unexpectedly, expression of *mei-218*^△^*^N^* in *mei-218* mutants (*mei-218*^△^*^N^*) restored crossing over to the same level as in *mei-218*; *mei-218^FL^* flies (55.9 cM; not significant, *P* = 0.61).

Brand *et al.* (2018) previously expressed *D. mauritiana* MEI-217 and MEI-218 in *D. melanogaster*, and found that crossovers were increased in proximal and distal regions, resulting in an overall change in crossover distribution. We examined crossover distribution in *mei-218*; *mei-218^FL^* and *mei-218*; *mei-218*^△^*^N^* flies (Figure 2C). Overall, distributions were similar, with both genotypes exhibiting strong inhibition of crossovers near the centromere (referred to as the centromere effect; Beadle 1932) and the majority of the crossovers being placed in the medial–distal regions (Figure 2C).

We conclude that the N-terminal tail of MEI-218 is dispensable for both crossover formation and overall distribution on chromosome 2L. This conclusion is supported by the observation that, of 16 sequenced mutations in *D. melanogaster mei-218*, 14 are nonsense or frameshift, and the only two missense mutations alter residues in the C-terminus (amino acids 845 and 1107) (Collins *et al.* 2012).

The reasons why the MCM domains have been separated into MEI-217 and MEI-218 polypeptides, and why MEI-218 has an N-terminal extension, are unknown, but this structure has been maintained for >250 MY of Dipteran evolution (Figure S2). Interestingly, the expression of MEI-218 is fairly high in testes (Thurmond *et al.* 2018), even though males do not experience meiotic recombination. The predominant or exclusive transcript in males does not encode MEI-217 (Thurmond *et al.* 2018), the seemingly obligate partner for MEI-218 in female meiotic recombination. Males that lack *mei-218* are viable, fertile, and do not exhibit elevated NDJ (Baker and Carpenter 1972; McKim *et al.* 1996). For these reasons, we speculate that an unknown function of MEI-218 (independent of MEI-217) in the male germline explains why its overall structure has been evolutionarily maintained.

### REC ATPase motifs are required for crossover formation

**Figure 3:**
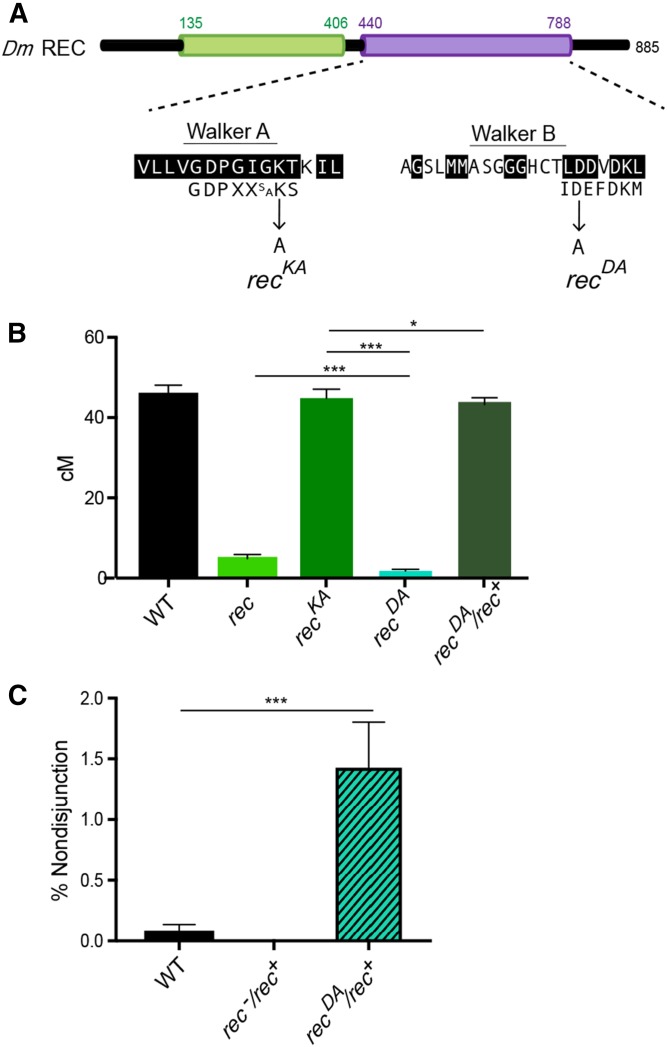
REC ATPase-binding and -hydrolysis requirements for crossover formation. (A) Schematic representation of the mutated residues in *rec^KA^* and *rec^DA^*. (B) Map units of WT (Hatkevich *et al.* 2017), *rec^1^/rec^2^*, *rec^KA^*, *rec^DA^*, and *rec^DA^/rec^+^*. Map units represented as centimorgans. Error bars show 95% C.I.s. (C) Percent nondisjunction of WT, *rec^1^/rec^+^*, and *rec^DA^/rec^+^*. * *P* < 0.05, ***P *<* 0.0001. Refer to Tables S2 and S3 for complete data sets. WT, wild-type.

Of the three known mei-MCM subunits, only REC harbors well-conserved Walker A and B motifs, suggesting that REC has ATP-binding and -hydrolysis activity (Kohl *et al.* 2012). It is unknown whether the mei-MCM complex utilizes REC’s putative ATPase activity for its function *in vivo*. To test this, we used clustered regularly interspaced short palindromic repeats/Cas9 to introduce mutations into *rec* that were predicted to disrupt the functions of the Walker A and B motifs (Figure 3A). The Walker A mutation (*rec^KA^*) results in the substitution of a conserved lysine residue with alanine; in other AAA+ ATPases, including replicative MCMs, this mutation prevents binding of ATP (Bell and Botchan 2013). The Walker B mutation (*rec^DA^*) results in the substitution of a conserved aspartic acid with alanine; in MCMs and other AAA+ ATPases, this mutation destroys the ability to coordinate Mg^++^ for ATP hydrolysis (Bochman *et al.* 2008).

We assayed crossover frequency along 2L in *rec^KA^* and *rec^DA^* mutants (Figure 3B). Surprisingly, *rec^KA^* ATP-binding mutants exhibit a genetic length of 44.9 cM, which is not significantly different from that of wild-type flies (*P* = 0.4016), suggesting that ATP binding by REC is not required for crossover formation. Conversely, there is a severe reduction in crossovers in *rec^DA^* mutants, with a genetic length of 1.6 cM (*P* < 0.0001), suggesting that REC’s ability to hydrolyze ATP is required for crossover formation.

Because the genetic length of *rec^DA^* is significantly lower than that of *rec* null mutants (Figure 3B, *P* < 0.0001), we hypothesized that *rec^DA^* is an antimorphic mutation. To test this, we examined crossover levels and *X* chromosome NDJ in *rec^DA^*/*rec^+^* (Figure 3, B and C, respectively). The genetic length of 2L in *rec^DA^*/*rec^+^* is slightly lower than in wild-type flies, but is not significantly different (43.9 and 45.8 cM, respectively; *P* = 0.35). For *X*-NDJ, both wild-type flies and *rec^−^/rec^+^* mutants exhibit rates < 0.5%, while *rec^DA^/rec^+^* mutants exhibit a significant increase to 1.4% NDJ (*P* < 0.0001). These data support the conclusion that *rec^DA^* is weakly antimorphic and suggest that *rec^DA^* results in an inactive mei-MCM complex that is antagonistic to the wild-type complex. In light of these interpretations, we propose that the mei-MCM complex binds to recombination sites independently of REC binding to ATP, and that REC-dependent ATP hydrolysis is required for the removal of the mei-MCM complex from these sites.

The phenotypes of *rec^KA^* and *rec^DA^* mutants suggest that REC’s ability to hydrolyze ATP is required for crossover formation, whereas its ATP-binding capability is dispensable. The disparate requirements for REC’s ATP binding and hydrolysis are similar to those of other ATPase-dependent complexes. RAD51 paralogs, which form multiprotein complexes and contain Walker A and B motifs, are proposed to exhibit ATPase activity in *trans* between adjacent subunits, each of which contributes a Walker A or Walker B motif to the active site (Wu *et al.* 2004, 2005; Wiese *et al.* 2006). Because neither MEI-217 nor MEI-218 possess an ATPase domain that harbors conserved key enzymatic residues (Figure 1B) (Kohl *et al.* 2012), we propose that the ATPase activity of the mei-MCM complex requires REC for ATP hydrolysis and an unknown mei-MCM protein for ATP binding. Alternatively, because REC is highly diverged, its Walker A and B motifs may function noncanonically. Biochemical studies are needed to test these hypotheses, but these may require the identification of the putative missing subunit.

### REC-dependent ATP hydrolysis is required for MEI-9-dependent crossovers

**Figure 4:**
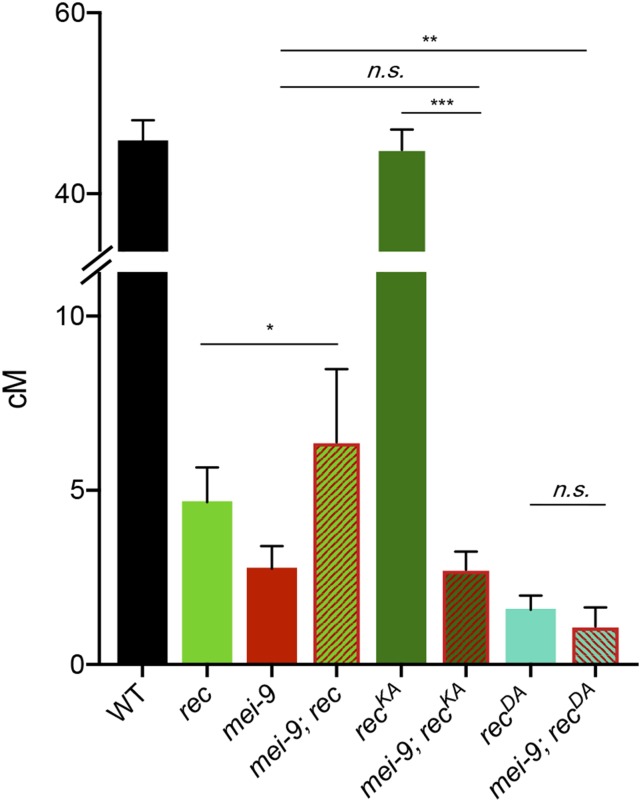
MEI-9-dependent crossovers in *rec^KA^* and *rec^DA^* mutants. Map units of WT (Hatkevich *et al.* 2017), *rec*, *mei-9*, *mei-9;rec*, *rec^KA^*, *mei-9;rec^KA^*, *rec^DA^*, and *mei-9;rec^DA^*. Map units represented as centimorgans. Error bars show 95% C.I.s. * *P* < 0.05, ** *P* < 0.001, *** *P* < 0.0001; (*mei-9* *vs.* *mei-9*; *rec^KA^*, *P* = 0.94) (*rec^DA^* *vs.* *mei-9*; *rec^DA^*, *P* = 0.23). Refer to Table S3 for complete data set. n.s., not significant; WT, wild-type.

To gain insight into the crossover pathways that are used in *rec^KA^* and *rec^DA^* mutants, we examined whether these crossovers require the Class I endonuclease/resolvase. In *Drosophila*, the catalytic subunit of the putative Class I meiosis-specific endonuclease is MEI-9 (Sekelsky *et al.* 1995; Yildiz *et al.* 2002; Hatkevich *et al.* 2017). The 2L genetic length within a *mei-9* mutant is 2.75 cM (Figure 4), demonstrating that ≥90% of crossovers are dependent upon MEI-9. However, the genetic length in *mei-9*; *rec* mutants is not significantly different from that of *rec* null single mutants (4.11 *vs.* 4.66 cM, *P* = 0.64), suggesting that, in the absence of REC, the resulting crossovers are likely independent of MEI-9. Similarly, it has been shown previously that *mei-218 mei-9* double mutants do not have reduced crossovers compared to *mei-218* single mutants (Sekelsky *et al.* 1995), indicating that crossovers generated in the absence of the mei-MCM complex are MEI-9-independent.

Because *rec^KA^* mutants exhibit the same distribution and number of crossovers as wild-type flies (Figure 3B), we hypothesized that *rec^KA^* crossovers are dependent on MEI-9. To test this, we examined genetic length across 2L in *mei-9*; *rec^KA^* double mutants (Figure 4). Mutants for *mei-9*; *rec^KA^* exhibited a genetic length of 2.72 cM, which was significantly decreased compared to the *rec^KA^* single mutant (*P* < 0.0001), but not significantly different from *mei-9* single mutants (*P* = 0.94), showing that crossovers in *rec^KA^* are indeed dependent upon MEI-9 nuclease. In contrast, we predicted that crossovers in *rec^DA^* would be independent of MEI-9, similar to crossovers generated in *rec* null mutants. We observed that *mei-9*; *rec^DA^* double mutants exhibit a genetic length of 1.1 cM, which is significantly lower than that of *mei-9* single mutants (*P* < 0.001). Importantly, crossing over in the *mei-9*; *rec^DA^* double mutant was not significantly different from in *rec^DA^* single mutants (*P* = 0.23), demonstrating that crossovers in *rec^DA^* are independent of MEI-9 (Figure 4).

From these data, we conclude that the crossovers in *rec^KA^* mutants arise through the normal, MEI-9-dependent pathway, whereas mitotic nucleases generate the residual crossovers in *rec^DA^* mutants. These data show that REC^KA^ functions normally in the Class I pathway, but that this pathway is nonfunctional in *rec* null and *rec^DA^* mutants. We suggest that the REC’s ability to hydrolyze, but not bind, ATP is required for the formation of Class I crossovers.

### REC ATPase motifs are required to prevent Class II crossovers

In wild-type *Drosophila*, most or all crossovers are generated through the Class I pathway (Hatkevich *et al.* 2017), and these crossovers are dependent upon the mei-MCM complex (Kohl *et al.* 2012). However, in *Blm* mutants, crossovers are generated exclusively through the Class II pathway (Hatkevich *et al.* 2017). In *Drosophila Blm* mutants, meiotic crossovers are decreased by 30%, suggesting that the Class II pathway is less efficient at generating crossovers than the Class I pathway, even though what may be the primary anticrossover protein, Blm helicase, is absent. It has previously been shown that loss of Blm suppresses the high NDJ of *mei-218* and *rec* mutants (Kohl *et al.* 2012). However, in *Blm rec* double mutants, crossovers are increased significantly compared to *Blm* single mutants (Kohl *et al.* 2012), suggesting that REC and/or the mei-MCM complex has an anticrossover role in *Blm* mutants, and therefore in the Class II crossover pathway.

To further understand the role of REC in the Class II pathway, we investigated whether REC’s predicted ATP-binding or -hydrolysis function is required for its Class II anticrossover function. To do this, we measured the crossovers across 2L in *rec^KA^* and *rec^DA^* in the background of *Blm* mutants. If REC ATP binding or hydrolysis is required for an anticrossover role in Class II, then the genetic length of *Blm rec^KA^* or *Blm rec^DA^* double mutants will be similar to that of *Blm rec* double mutants. Conversely, if REC ATP binding or hydrolysis is not required, then double mutants will exhibit genetic lengths similar to that of *Blm* single mutants.

**Figure 5:**
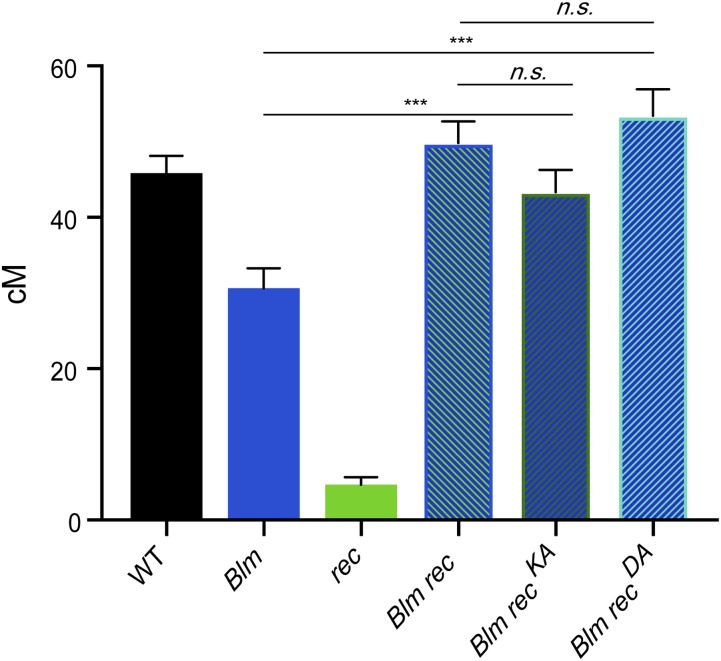
Requirements of REC ATPase activity in Blm function. Map units of WT (Hatkevich *et al.* 2017), *Blm* (Kohl *et al.* 2012), *rec*, *Blm rec* (Kohl *et al.* 2012), *Blm rec^KA^*, and *Blm rec^DA^*. Map units represented as centimorgans. Error bars show 95% C.I.s. Refer to Table S3 for complete data set. *** *P* < 0.0001 (*Blm rec* *vs.* *Blm rec^KA^*, *P* = 0.10) (*Blm rec* *vs.* *Blm rec^DA^*, *P* = 0.52). n.s., not significant; WT, wild-type.

Interestingly, *Blm rec^KA^* mutants exhibit a genetic length of 43.3 cM, which is not significantly different from that of *Blm rec* mutants (*P* = 0.10) but is significantly higher than that of *Blm* single mutants (*P* < 0.0001; Figure 5). Similarly, *Blm rec^DA^* double mutants have a recombination rate of 53.4 cM, which is not significantly different from *Blm rec* double mutants (*P* = 0.52), but is significantly higher than that of *Blm* single mutants (*P* < 0.0001). These results suggest that REC’s predicted abilities to bind and hydrolyze ATP are both required for the inhibition of crossovers at REC-associated Class II recombination sites. Therefore, it appears that REC forms different complexes within the Class II and Class I pathways. It is unknown whether this Class II REC-associated complex requires the other mei-MCM proteins, and additional genetic studies will be valuable to discern this.

In summary, the mei-MCMs are a family of diverged proteins that help to establish the recombination landscape in *D. melanogaster* by promoting Class I crossovers and inhibiting Class II crossovers. Results obtained in this study have further elucidated meiotic recombination roles for two mei-MCM proteins, MEI-218 and REC. While the N-terminus of MEI-218 is dispensable for crossover formation (Figure 2), REC’s predicted ability to bind and hydrolyze ATP exhibits differential requirements for the regulation of Class I and Class II crossover formation. From our genetic analyses, we suggest that the Walker B motif of REC, but not the Walker A motif, is required for promoting the formation of Class I, MEI-9-dependent crossovers (Figure 3 and Figure 4). The weakly antimorphic phenotype of *rec^DA^* demonstrates that an impaired REC Walker B mutant renders a poisonous complex; a complex that we propose cannot be released from recombination sites. Both Walker A and Walker B motifs block crossovers in the Class II pathway, suggesting that REC forms different complexes to execute its pro- and anticrossover functions. Biochemical and cytological studies are needed to support or refute these hypotheses.
